# High-Throughput Docking Using Quantum Mechanical Scoring

**DOI:** 10.3389/fchem.2020.00246

**Published:** 2020-04-21

**Authors:** Claudio N. Cavasotto, M. Gabriela Aucar

**Affiliations:** ^1^Computational Drug Design and Biomedical Informatics Laboratory, Translational Medicine Research Institute (IIMT), CONICET-Universidad Austral, Pilar, Argentina; ^2^Facultad de Ciencias Biomédicas and Facultad de Ingeniería, Universidad Austral, Pilar, Argentina; ^3^Austral Institute for Applied Artificial Intelligence, Universidad Austral, Pilar, Argentina

**Keywords:** high-throughput docking, structure-based drug design, molecular docking, quantum mechanics, semi-empirical methods

## Abstract

Today high-throughput docking is one of the most commonly used computational tools in drug lead discovery. While there has been an impressive methodological improvement in docking accuracy, docking scoring still remains an open challenge. Most docking programs are rooted in classical molecular mechanics. However, to better characterize protein-ligand interactions, the use of a more accurate quantum mechanical (QM) description would be necessary. In this work, we introduce a QM-based docking scoring function for high-throughput docking and evaluate it on 10 protein systems belonging to diverse protein families, and with different binding site characteristics. Outstanding results were obtained, with our QM scoring function displaying much higher enrichment (screening power) than a traditional docking method. It is acknowledged that developments in quantum mechanics theory, algorithms and computer hardware throughout the upcoming years will allow semi-empirical (or low-cost) quantum mechanical methods to slowly replace force-field calculations. It is thus urgently needed to develop and validate novel quantum mechanical-based scoring functions for high-throughput docking toward more accurate methods for the identification and optimization of modulators of pharmaceutically relevant targets.

## Introduction

The cost to bring a new drug to the market could be as high as 2.6 billion US dollars, and can take up to 15 years (DiMasi et al., 2016). For many years, both the identification and optimization of novel drug lead compounds were accomplished within the drug discovery process by the experimental high-throughput screening of large chemical libraries. In spite of multiple efforts to improve its performance, drug discovery remains a costly and time consuming technique (Phatak et al., 2009). However, for the last 25 years, theoretical developments, better computational algorithms, faster computing resources, and improved visualization tools enabled the routine use of computational methods to model and visualize protein-ligand (PL) interactions, calculate binding free energy to different degrees of accuracy, and *in silico* screen chemical libraries using ligand-based and structure-based approaches. Today, computational chemistry is firmly established as a valuable tool in any drug lead discovery endeavor, aimed at saving time, effort, resources, and reducing costs (Cavasotto and Orry, 2007; Jorgensen, 2009, 2012; Spyrakis and Cavasotto, 2015; Pagadala et al., 2017).

During the last three decades, molecular docking has been one of the most commonly used computational methods in drug lead discovery (for review, cf., Kitchen et al., 2004; Rognan, 2011; Ciancetta and Moro, 2015; Sotriffer, 2015; Spyrakis and Cavasotto, 2015; Sulimov et al., 2019b). The aim of protein-small-molecule docking is the characterization of the optimal binding modes (poses) of a molecule within the binding site, and an estimation of its binding free energy. In high-throughput docking (HTD), where the protein is usually considered rigid or with very few degrees of freedom, and thousands to millions of molecules from a chemical library are screened, the goal is to generate a sub-library enriched with potential ligands, which will be prioritized for further experimental evaluation. In HTD, two different stages can be distinguished: the assessment of the best binding mode(s) of each molecule of the library (“docking stage”), and, on each *in silico* generated protein-small-molecule complex, the calculation of a score reflective of the likelihood that the molecule will actually bind to the target (“scoring stage”) (Cavasotto and Orry, 2007; Guedes et al., 2018). In the docking stage, the docking energy (DE) is used to select, for each molecule, the lowest-energy pose(s) from a large amount of conformations generated, while the docking score (DS) is generally calculated as a fast approximation to the binding free energy (Δ*G*_*binding*_), and depends on several factors, such as the energy representation of the system, the model used to represent the aqueous environment and the consideration of explicit water molecules within the active site (Cozzini et al., 2006; Amadasi et al., 2008), and the degree of consideration of receptor flexibility (Cavasotto and Singh, 2008; Spyrakis et al., 2011; Spyrakis and Cavasotto, 2015). Thus, DE discriminates among poses of the same molecule, while the DS characterizes each molecule of the docked chemical library and is used to rank them according to the likelihood of binding. Many docking programs, however, use a single function as DE and DS.

It should be stressed that one of the main advantages of docking is that *in silico* generated poses usually serve as the starting point for *in silico* ligand optimization, using for example molecular dynamics-based calculation of binding free energies, such as Molecular Mechanics-Poisson Boltzmann Surface Area (MM-PBSA) and MM-Generalized Born Surface Area (MM-GBSA) methods (Kerrigan, 2013; Reddy et al., 2014; Genheden and Ryde, 2015; Sun et al., 2018; Wang et al., 2019).

While docking accuracy depends on the program, it is acknowledged that most of them are usually successful in identifying the correct pose (RMSD < 2 Å) with respect to the native structure (Warren et al., 2006; Wang et al., 2016). Moreover, an extensive recent benchmark of the Comparative Assessment of Scoring Functions (CASF) (Su et al., 2019) highlighted that docking programs display a better performance in terms of docking accuracy than in any of these three scoring-related metrics: correlation with experimental binding data (scoring power), ranking of ligands by their binding affinity data provided their correct poses are known (ranking power), and identification of actual ligands from a sub-library of top-ranking small-molecules (screening power). This was in agreement with other works (Cavasotto and Abagyan, 2004; Slater and Kontoyianni, 2019).

Most docking developments have been mainly rooted in molecular mechanics (MM) force-fields (FF). However, to better characterize protein-ligand interactions, at least in some cases, the use of a quantum mechanical (QM) description would be necessary (Cavasotto et al., 2019). The QM formulation is theoretically exact, as in principle, it accounts for all contributions to the energy (including terms or effects usually missing in FFs, such as electronic polarization, charge transfer, halogen bonding, and covalent-bond formation). Moreover, the QM framework is general across the chemical space so that all elements and interactions can be considered on equal footing, thus avoiding MM parameterizations.

Following the pioneering work of Raha and Merz (2004, 2005) where a QM-based score was used to discriminate ligand from decoy poses, there have been recently some applications of QM methods in docking, mainly aiming for accurate ligand binding mode assessment (for a survey of recent related works cf., Mucs and Bryce, 2013; Cavasotto et al., 2018; Aucar and Cavasotto, 2020). In a significant step forward, Pecina et al. obtained impressive results on the discrimination of native from decoy docking poses on four challenging systems (Pecina et al., 2016) using a docking energy function (Lepšík et al., 2013) based on the semi-empirical QM PM6 Hamiltonian (Stewart, 2007) supplemented with the D3H4X correction for dispersion, hydrogen- and halogen-bonding interactions (Rezáč and Hobza, 2012). In a follow-up contribution (Pecina et al., 2017), an even superior performance was achieved for accurate pose assessment using a self-consistent-charge density-functional tight-binding method (SCC-DFTB) formulation coupled with D3H4 corrections for dispersion and hydrogen-bond interactions, though at a higher computational cost. This docking energy score function was further used to obtain a reliable ranking on 10 inhibitors binding to carbonic anhydrase II (CAII) (Pecina et al., 2018).

However, the development of QM-based docking scoring functions aiming at the ranking of molecules within HTD (screening power) has progressed at a significantly slower pace. Only very recently, a QM-based approach was presented displaying a very good performance on discriminating ligands and decoys on a single system (heat shock protein 90, HSP90) (Eyrilmez et al., 2019). In fact, the development of fast yet accurate docking scoring functions still constitutes an area of active research (Cavasotto, 2012; Guedes et al., 2018). Moreover, the blind challenges ran by the Drug Design Data Resource (D3R) for ligand-pose and affinity prediction in 2015 (Gathiaka et al., 2016), 2016 (Gaieb et al., 2018), and 2018 (Gaieb et al., 2019), have shown the importance of method development and benchmarking in pose prediction and binding affinity ranking of ligands.

In this work, we introduce a QM-based docking scoring function and evaluate it in terms of ligand enrichment on 10 protein systems belonging to diverse protein families in terms of different binding site characteristics, the presence of co-factors and water molecules, and the enrichment factors computed with a standard HTD method. Excellent results were obtained by displaying our QM-based scoring function a much higher enrichment (screening power) than a traditional docking method. We stress that our goal is to present and to validate an initial straightforward approach, which could serve as a starting point for further developments and improvement. A wider and extensive benchmarking on more systems and a systematic comparison with most of the standard docking programs, and the assessment of the optimal combination of the different components of our approach (QM formalism and continuum solvent model, energy minimization strategies, use of single or multiple docking poses for scoring, and entropy contribution) are considerations of their importance. However, they exceed the purpose of our work and will be published in due course.

Assuming a continuous development in QM theory, algorithms and computer hardware, it is likely that semi-empirical methods [or low-cost Density Functional Theory (DFT) methods] will replace FF over the next 25 years (Grimme and Schreiner, 2018). Therefore, it is absolutely justified and there is an urgent need to start developing the next generation of QM-based scoring functions for HTD toward better methods for the identification of small-molecule modulators of pharmaceutically relevant targets.

## Materials and Methods

### Protein Systems Preparation

The following targets were downloaded from the PDB (cf. Table 1): Cyclin-dependent Kinase 2 (CDK2, PDB 1FVV), Estrogen Receptor α (ESR1, PDB 3ERT), Cyclooxygenase-1 (COX1, PDB 2OYU), Neuraminidase (NRAM, PDB 1B9V), Heat Shock Protein 90 α (HSP90a, PDB 1UYG), Hexokinase Type IV (HXK4, PDB 3F9M), Coagulation Factor VII (FA7, PDB 1W7X), Thymidine kinase (KITH, PDB 2B8T), Fatty Acid Binding Protein Adipocyte (FABP4, PDB 2NNQ), and Phospholipase A2 (PA2GA, PDB 1KVO). All water molecules and co-factors were deleted, except in the following cases: NRAM and PA2GA, the Ca^2+^ atom within 8 Å of the bound ligand; HSP90a, water molecules 2059, 2121, 2123, and 2236; FA7, water molecule 2440; FABP4 water molecules 303, 623, 634, 665.

**Table 1:** Target proteins used in the evaluation of QM-based scoring functions.

**Receptor name**	**Receptor code**	**PDB code**	**Co-factor^***a***^**	**Number of water molecules^***b***^**	***EF*(1)^***c***^**
Cyclin-dependent Kinase 2	CDK2	1FVV	–	–	8.0
Estrogen receptor α	ESR1	3ERT	–	–	16.5
Cyclooxygenase-1	COX1	2OYU	–	–	1.3
Neuraminidase	NRAM	1B9V	Ca^2+^	–	0.
Heat shock protein 90 α	HSP90a	1UYG	–	4	0.
Hexokinase type IV	HXK4	3F9M	–	–	1.1
Coagulation factor VII	FA7	1W7X	–	1	20.2
Thymidine kinase	KITH	2B8T	–	–	35.1
Fatty acid binding protein adipocyte	FABP4	2NNQ	–	4	31.9
Phospholipase A2	PA2GA	1KVO	Ca^2+^	–	2.0

a *Within 8 Å of the cyrstallographic ligand*.

b *Within 4 Å of the cyrstallographic ligand*.

c *Enrichment factor at 1% corresponding to docking with AutoDock Vina*.

Each target was prepared using ICM software (MolSoft, San Diego, CA, 2019; Abagyan et al., 1994) in a similar fashion as in earlier works (Phatak et al., 2010). Succinctly, hydrogen atoms were added, followed by a local energy minimization of the complete system, and polar and water hydrogen positions were determined by optimizing the hydrogen bonding network within the torsional coordinates space. All Asp and Glu residues were assigned a −1 charge, and all Arg and Lys residues were assigned a +1 charge. Histidine tautomers were chosen according to their corresponding hydrogen bonding pattern. For docking with AutoDock Vina (Trott and Olson, 2010), the systems were pre-processed with AutoDock Tools (Morris et al., 2009).

### Docking Library Preparation

For each target, the docking libraries were built by merging a set of ligands and a set of decoys, where the latter had similar physico-chemical properties to the ligands, but dissimilar 2-D topology. This has been shown to be necessary to ensure unbiased results when benchmarking docking programs (Huang et al., 2006; Gatica and Cavasotto, 2012). Ligands and decoys were extracted from the Directory of Useful Decoys (DUD, Huang et al., 2006), the NRLiSt binding data base for nuclear receptors (Lagarde et al., 2014), or the Directory of Useful Decoys- Enhanced (DUD-E, Mysinger et al., 2012), according to: CDK2, DUD (72, 2074) (number of ligands, number of decoys); ESR1, NRLiSt (133, 6555); COX1, DUD-E (210, 6955); NRAM, DUD-E (222, 6227); HSP90a (125, 4942); HXK4, DUD-E (127, 4802); FA7, DUD-E (185, 6300); KITH, DUD-E (132, 2866); FABP4, DUD-E (57, 2855); PA2GA (127, 5215). The protonation state and chirality of all molecules were conserved as in their original database.

### High-Throughput Docking With AutoDock Vina

Molecular docking of the chemical libraries onto the associated targets using AutoDock Vina (Trott and Olson, 2010) was performed in a similar fashion as in our recent work (Palacio-Rodriguez et al., 2019).

### Protein-Molecule Complex Generation, Structural Relaxation, and Unbound Protein and Ligand States Characterization

Protein-molecule complexes for QM-scoring were generated using the ICM docking module, keeping for each molecule its lowest DE conformation (docking RMSD values of native ligands are shown in Table 2). These protein-molecule complexes were also relaxed through cycles of local energy minimization in ICM according to the following procedure: (i) For each protein, the collected dihedral angles of amino-acids within 4 Å of any docked ligand of the corresponding chemical library were considered free; (ii) For each protein-molecule complex, five cycles of local energy minimization were performed restraining the heavy atoms with a harmonic potential with respect to their initial conformation; in each cycle the weight of this added potential was reduced in the following way: 50, 10, 5, 1, and 0 kcal/mol (no restraint). During this local energy minimization, the protein system was optimized in the torsional space (Abagyan et al., 1994), and the small-molecule in the Cartesian space.

**Table 2:** RMSD values of docked native ligands.

**Receptor name**	**Receptor code**	**PDB ligand ID**	**RMSD** **(Å)**	
			**ICM**	**AD Vina**
Cyclin-dependent Kinase 2	CDK2	107	0.74	2.68
Estrogen receptor α	ESR1	oht	1.48	4.69
Cyclooxygenase-1	COX1	ims	1.40	0.36
Neuraminidase	NRAM	ra2	0.54	0.91
Heat shock protein 90 α	HSP90a	pu2	0.52	0.26
Hexokinase type IV	HXK4	mrk	0.47	7.56
Coagulation factor VII	FA7	413	0.31	0.60
Thymidine kinase	KITH	thm	0.22	0.70
Fatty acid binding protein adipocyte	FABP4	t4b	0.37	0.79
Phospholipase A2	PA2GA	oap	0.54	1.37

To generate the unbound states, local energy minimization was performed on both protein and small-molecule in isolation from the crystallographic structure and the docked conformation, respectively.

### System Cutout

For each target, a reduced-system was defined by first listing all the amino-acids within 8 Å of any docked molecule with ICM (only heavy atoms were considered in this threshold). Then, upon visual inspection, other amino-acids were eventually added to the list in order to avoid intra-helix or intra-β-sheet fragmentation, or loop fragments with just one amino-acid. A reduced-system was then built by deleting from the structure all amino-acids not included in the list, capping the N- and C-terminal of each fragment with hydrogens.

### Entropy Calculation

Binding small-molecule conformational entropy was estimated as

(1) $$\begin{array}{l} \Delta S=-R\ln\;\Omega \end{array}$$

where it is assumed that, upon binding, the molecule adopts a single conformation state (thus *S*_*bound*_ = 0), and Ω is the number of conformations in the free state, which was estimated in two different ways: i) by assigning each of the *N* free torsional bonds three rotational degrees of freedom (and thus Ω = 3^*N*^); ii) by performing a Monte-Carlo (MC) sampling with local energy minimization in the torsional space using ICM (Abagyan and Totrov, 1994; Abagyan et al., 1994), collecting all distinct conformations within the lowest 3 kcal/mol energy, and assuming all conformers are equally probable (a similar low-level sampling approach was used to explore the conformational flexibility of small-molecules, Forti et al., 2012). The MC approach was considered since rotamer count is known to over-estimate the number of low-energy conformations, and thus the entropy (Anisimov and Cavasotto, 2011).

### Quantum Mechanical Calculations

All QM calculations were performed using the QM package MOPAC2016 (Stewart, 2016) and its linear-scaling module MOZYME (Stewart, 1996), using the semi-empirical PM7 Hamiltonian (Stewart, 2013). In agreement with other authors (Sulimov et al., 2017a), we selected PM7 since it accounts for dispersion interactions, and hydrogen and halogen bonding have been taken into consideration at the paramterization stage, while it also includes several corrections to the PM6 Hamiltonian. Moreover, PM7 exhibited a very good performance on energy calculations aimed at discriminating native ligand positions in crystallographic complexes (Sulimov et al., 2017b). The solvation energy contribution in aqueous environment was calculated using the Conductor Like Screening Model (COSMO, Klamt and Schüürmann, 1993) continuum solvent model, with default atomic radii and surface tension parameters. The solvent-accessible surface area was taken from the program output [cf. (Stewart, 2016) for details on how the surface is built]. Those molecules which did not complete the QM calculation were excluded when computing the enrichment.

### Evaluation Metrics

The enrichment factor (*EF*) measures the enrichment of actual ligands in a docked hit-list given a specific percentage of the dataset (threshold). The *EF* is defined as the ratio between actual number ligands (hits) found at the top *x*% of the screened database (*Hits*_*x*__%_) and the number of molecules at that threshold *N*_*x*__%_, normalized by the ratio between the total number of actual ligands within the entire dataset (*Hits*_total_) and the total number of molecules of the latter (*N*_total_).

(2) $$\begin{array}{l} EF\left(x\right)=\frac{Hits_{x\%}}{N_{x\%}}/\frac{Hits_{total}}{N_{total}} \end{array}$$

Thus, the EF represents the probability of finding an actual ligand within the *x*% of the screened database with respect to the probability of finding an actual ligand at random. Whenever a molecule is represented within a chemical library with different states according to its protonation or chirality, each state is assigned an individual score, and the lowest score is used in the hit-list, and thus to calculate the *EF*. Throughout this work we report *EF*(1) and *EF*(2), since they are more representative of early enrichment.

We also report receiver operating characteristics (ROC) curves for each of the studied systems, measuring the area under the curve (AUC).

## Theoretical Framework

The binding free energy (Δ*G*_*binding*_) corresponding to Protein-Ligand (PL) association is expressed within the end-point molecular mechanics-quantum mechanics surface area method (MM-QMSA) (Anisimov and Cavasotto, 2011; Anisimov et al., 2011) as

(3) $$\begin{array}{l} \Delta G_{binding}=\Delta \langle G^{QM}\rangle -T\Delta S \end{array}$$

where the difference in the first term is calculated between the bound (PL) and unbound (P, L) states, <…> represents the average over QM-minimized classical molecular dynamics (MD) trajectories, *G*^*QM*^ is the QM energy including a continuum solvation term in an aqueous environment, and the second term represents the entropy change of P and L upon binding. We prefer to note the first term as a free energy, since it also includes the change in solvation free energy.

Since Δ*G*_*binding*_ in Equation (3) is obviously too costly to be used to score and rank large chemical libraries of small-molecules in HTD, a reasonable QM docking scoring function (*QMDS*) can be defined as an approximation to Equation (3), namely

(4) $$\begin{array}{l} QMDS=\Delta G^{QM}-T\Delta S \end{array}$$

where averages over MD trajectories have been replaced by single-point QM calculations on the docked PL structure, and the free unbound L and P structures. The L and P deformation penalty contributions due to changes in L and P conformations upon binding are expressed as

(5) $$\begin{array}{l} \Delta G_{conf}^{QM}\left(X\right)=G_{o}^{QM}\left(X\right)-G^{QM}\left(X\right)\;,\text{ with }X=\text{L},\text{P} \end{array}$$

where *G*_*o*_(X) is the energy of the isolated X in the conformation of the docked PL complex, and *G*(X) is the energy of X in the free unbound state. Considering Equation (5), Equation (4) can be now be written out making the deformation contributions explicit as

(6) $$\begin{array}{l} QMDS=\Delta G_{o}^{QM}+\Delta G_{conf}^{QM}\left(P\right)+\Delta G_{conf}^{QM}\left(L\right)-T\Delta S \end{array}$$

where the “o” subscript in the first term refers to calculations using the PL, P, and L conformations from the docked complex. It should be pointed out that Equation 6 is formally identical to another formulation (Eyrilmez et al., 2019).

Two types of QM docking scoring functions were defined according to the relaxation of the reference docked PL complexes: (i) *QMDS*_1_, with no relaxation, that is, the QM calculations are performed directly on the docked PL complex, and (ii) *QMDS*_2_, where docked PL complexes are relaxed through local energy minimization (see Methods). When the deformation contributions (second and third terms in Equation 6) were included, the suffix “d” is added (*QMDS*_1*d*_ and *QMDS*_2*d*_).

## Results and Discussion

### Improved HTD Enrichment Using QM-Based Scoring

Ten target proteins were selected based on different characteristics such as protein family, binding site properties, presence of co-factors and water molecules (within or close to the binding site), and enrichment factor at 1% calculated after docking with AutoDock Vina (Table 1). Only crystallographic and/or conserved water molecules within 4 Å of the native ligand were included.

Throughout all this work, the *QMDS* was calculated in all its variants on PL complexes generated with ICM docking, since it is acknowledged to generate high quality protein-molecule poses (Bursulaya et al., 2003; Neves et al., 2012), as confirmed by the RMSD values of the docked native ligands in Table 2). Clearly, better enrichment is strongly coupled to scoring over correct docking poses. In this regard, the use of multiple docked conformations for each molecule, stemming from the same docking program or not, might clearly enhance the results of our QM-scoring scheme. However, we preferred to use a single pose from a single program, to keep our methodology straightforward, and to establish a clear baseline from which to start looking for improvement.

Since a target receptor protein is usually very large for QM calculations, to calculate the *QMDS* we used a reduced system by cutting out amino acids farther than ~8 Å from any docked molecule (cf. the Methods section for full details on the cutout process), since a threshold of <6 Å has been reported to seriously deteriorate the results (Ehrlich et al., 2017); moreover, it should be highlighted that the smaller the threshold, the greater the impact of the continuous solvent surface replacing the cutout amino-acids. To further validate our approach, quantum mechanical docking scores *QMDS* considering the complete protein and its associated reduced system were calculated on CDK2 and ESR1 (Table 3). We observe that using a cutout system has no impact on the calculation. Thus, throughout this work, a reduced representation of the target protein will be used for all QM calculations.

**Table 3:** Comparison of the enrichment factors [*EF*(1)] for docking and scoring (*QMDS*_1_) using a complete and reduced protein systems.

**Receptor**	**Complete system**	**Reduced system**
CDK2	20.1	24.2
ESR1	33.7	36.4

In Table 4, we display the enrichment factors *EF*(1) for the 10 target systems comparing AutoDock Vina with four schemes of QM docking scoring (for HSP90a, enrichment values including and excluding the 19 macrocycle containing ligands are shown). The conformational entropy change upon ligand binding was estimated in two ways: (i) Δ*S*^*rot*^, based on a term proportional to the number of *N* free rotatable bonds of the molecule (Ω_*conf*_ = 3^*N*^), and (ii) Δ*S*^*conf*^, by estimating Ω_*conf*_ as the number of low-energy diverse conformations generated using Monte-Carlo sampling with local energy minimization (cf. Methods). We found that the use of *S*^*rot*^ deteriorates the *EF* (data not shown), so *S*^*conf*^ is used in all calculations. In *QMDS*_2_ and *QMDS*_2*d*_ the reference docked PL complexes were local energy minimized using MM (see Methods). Obviously, a QM minimization would have been desirable, but this would render any QM docking scoring function useless due to the computational times involved, even for reduced systems. Moreover, in this case further caution should be exerted not to artificially deform the molecular system.

**Table 4:** Enrichment factors calculated at 1% [*EF*(1)] and 2% [*EF*(2)] (in parenthesis) for AutoDock Vina and QM docking scoring.

**Receptor**	**AD Vina**	***QMDS_**1**_***	***QMDS_**1*d***_***	***QMDS_**2**_***	***QMDS_**2*d***_***
CDK2	8.0(5.0)	24.2(15.1)	26.2(18.1)	26.3(16.2)	26.3(18.2)
ESR1	16.5(11.0)	36.4(26.1)	30.6(22.4)	44.0(29.2)	43.2(26.4)
COX1	1.3(0.7)	2.8(2.8)	3.5(3.9)	2.8(1.4)	3.5(3.5)
NRAM	0. (0.)	9.3(8.7)	9.3(10.2)	21.4(15.8)	21.4(16.3)
HSP90a	0. (0.)	15.3(11.9)	16.7(11.9)	28.3(16.1)	31.1(16.1)
HSP90a^a^	0. (0.)	14.1(10.5)	15.2(10.5)	23.3(14.5)	26.8(14.0)
HXK4	1.1(1.1)	11.4(7.9)	9.1(6.2)	15.6(8.8)	15.6(9.9)
FA7	20.2(20.2)	49.0(42.5)	47.0(42.5)	54.0(41.0)	52.0(41.0)
KITH	35.1(21.1)	35.4(23.8)	31.7(22.9)	34.1(24.2)	30.5(26.9)
FABP4	31.9(16.0)	33.9(18.9)	36.2(20.9)	32.8(18.6)	32.8(23.0)
PA2GA	2.0(2.0)	6.5(7.5)	10.9(8.6)	18.2(12.9)	16.1(12.9)

a *Excluding the macrocycle containing molecules for calculating the EF*.

As stated before, a wide range of enrichment factors calculated from docking with AD Vina was taken into account for selecting the target proteins for this benchmark. It can be readily seen from Table 4 that using any variant of QM docking scoring has an impressive improvement over AD Vina, especially in those cases with low AD Vina *EF*. This happens even in the simplest case of *QMDS*_1_, where no relaxation is performed on the PL complexes.

It is clear that PL relaxation, even using a MM-based approach, has on average a positive effect for calculating the QM docking score. Moreover, in those cases where the *EF*(1) slightly decreases (KITH, FABP4), the *EF*(2) is conserved. Focusing in the analysis of *QMDS*_2_ and *QMDS*_2*d*_, inclusion of the deformation contribution (second and third term in Equation 6) slightly deteriorates the results in ESR1, FA7, KITH, and PA2GA. However, in all but ESR1, *EF*(2) improves after inclusion of the deformation term (as it also happens in the other cases where *EF*(1) increases or is constant, CDK2, COX1, NRAM, HSP90a, HXK4, and FABP4). Considering that the effect on *EF*(1) is in no way dramatic, and that *EF*(2) (which also refers to early enrichment), improves except in one case, we state that the deformation terms are necessary to obtain better enrichment factors, though this should obviously be validated in a larger-scale benchmark. We hypothesize that this slight deterioration might be related to a small noise introduced upon energy minimization, which is canceled out in the *QMDS*_2_ case. In the special case of HSP90a, the consideration of 19 macrocycle containing molecules has a negative effect in the *EF* calculation. We hypothesize that the strong performance of QM-scoring is due to a better representation of intra- and inter-molecular interactions, though of course further validation and benchmarking is still needed to confirm this.

**Figure 1:**
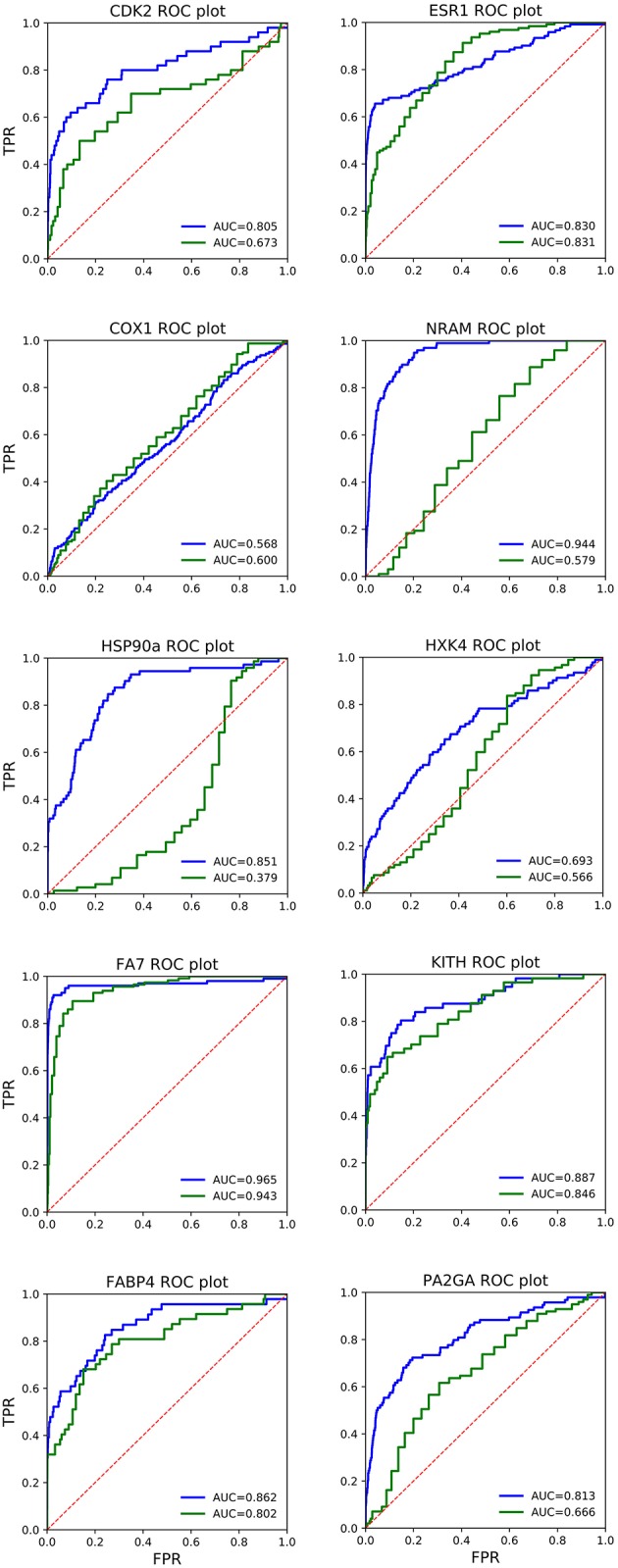
Receiver operating characteristic (ROC) plots of AutoDock Vina (red line) and QM-scoring *QMDS*_2*d*_ (blue line) for the 10 systems studied. The dotted line corresponds to random selection (AUC = 0.5). FPR, False Positive Rate; TPR, True Positive Rate; AUC, Area under the curve.

**Figure 2:**
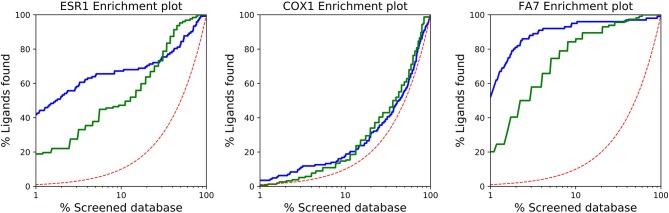
Enrichment plots for ESR1, COX1, and FA7 using AutoDock Vina (red line) and QM-scoring QMDS_2d_ (blue line). The dotted line corresponds to random selection.

In Figure 1, the ROC plots of *QMDS*_2_ and AD Vina for the 10 systems are shown, including the corresponding AUC values. Analysis of the curves confirm what has been noted above based on *EF*, exhibiting the QM-score excellent results. Interestingly, in ESR1 both scoring methods show basically the same AUC, which is in conflict with the large difference in EF values reported in Table 4. To clarify this issue, in Figure 2 we show the enrichment plot associated to ESR1. It can be seen that AD out-performs *QMDS*_2_ after 30% of the screened database, a region of no importance for drug discovery; for early enrichment, the enrichment plot in Figure 2 confirms the trend observed in Table 4 that *QMDS*_2_ is remarkable superior in the initial part of the ranking. A similar behavior is observed for FA7 (cf. Table 4 and Figures 1, 2). In the case of COX1, while the AUC of the QM-score is slightly less than AD Vina, the enrichment plot in Figure 2 shows that for early enrichment, QM-scoring out-performs AD Vina.

While our QM-score appears to be a very promising for HTD, and QM calculations are in principle more accurate than classical ones to describe molecular interactions, there are still a number of approximations which prevent the direct use of *QMDS* as a measure of actual absolute binding free energy. We mention three, among many: (i) QM local energy minimization was not performed (for computational efficiency, as said above); (ii) Vibrational entropies were not included; (iii) PM7 has not been parameterized to reproduce binding free energies. Our QM calculations were in the order of −70 kcal/mol, in agreement with recent binding enthalpy calculations on protein-ligand complexes using a PM7+COSMO approach (Sulimov et al., 2019a), where in spite of the difference between experimental and calculated absolute binding enthalpies, very good correlation with experimental values was obtained. It should be added that it is also well-known that traditional scoring functions correlate poorly with binding energy (cf. Enyedy and Egan, 2008, among others). Moreover, among traditional scoring functions there is no uniform scale: While AutoDock and Glide (Friesner et al., 2004; Halgren et al., 2004) are roughly in the range of −10 kcal/mol and higher, others are around −60 kcal/mol. Moreover, even end-point methods such as MM/PBSA or MM/GBSA exhibited calculated binding free energies in the order of −60 kcal/mol, or even lower when changes in vibrational entropy are not included (Zhong and Carlson, 2005), and even when including those terms (Woo and Roux, 2005; Anisimov and Cavasotto, 2011; Anisimov et al., 2011). Thus, we stress that *QMDS* should be considered a score, not a measure of absolute binding energy. It is aimed for relative binding energy estimation, and thus for compound ranking.

On average, the computing time of this QM docking score on a single core is ~6–8 minutes (depending on the size of the system, and on whether the deformation energy term is considered), around an order of magnitude slower than a MM-based DS.

## Conclusions and Perspectives

Docking programs have been so far based on molecular mechanics force-fields. However, a better description of protein-ligand interactions could be achieved, in principle, with quantum mechanical methods, which are theoretically exact, capture the underlying physics of the molecular system, and account for all contributions to the energy, including those effects usually missing in force-fields, such as electronic polarization, covalent-bond formation, and charge transfer. Moreover, a quantum mechanical formulation is generally valid across the chemical space, thus avoiding the force-field parameterizations.

We present a new QM-based high-throughput docking scoring function, which has been evaluated on 10 protein systems belonging to different protein families, displaying diverse binding site properties, and covering a wide range of enrichment factors computed with a traditional docking program. As shown in Table 4, even the simplest QM docking scoring function (where no relaxation is performed on the reference docked protein-small-molecule complex) shows excellent results in terms of enrichment (screening power). In fact, the improvement over AutoDock Vina on all systems is remarkable, especially in those cases with very low AD Vina enrichment. Upon complex relaxation, the improvement is even larger, regardless of whether the protein and ligand deformation terms are included or not.

We highlight that our main aim is to develop and validate a simple, straightforward approach for QM docking scoring, from which further developments can be built. Clearly, to further improve this methodology, several aspects should be analyzed: (i) a wider and extensive benchmark on many more target systems; (ii) comparison with other MM-based standard docking scoring functions; (iii) evaluation of other QM formalisms, continuum solvent models and their associated parameters (atomic radii and surface tension parameters); (iv) structural relaxation strategies; (v) use of single or multiple poses for scoring; (vi) the vibrational entropy changes upon binding. All of these considerations are important. They are currently being investigated and will be published in due course. Considering the outstanding improvements to our methods, we highlight that the *QMDS* should be used as a score and not an estimation to the absolute binding energy.

In terms of CPU time, our QM docking scoring function is approximately 10 times slower than MM-based standard scores on a single core. In spite of this, our impressive results on a set of 10 different protein targets highlight the huge potential of QM-based scoring. Moreover, considering future developments in QM theory, algorithms and computer hardware, it can be hypothesized that semi-empirical methods (or low-cost DFT methods) will replace FF over the following years (Grimme and Schreiner, 2018). We thus believe it is fully justified and of the utmost importance to develop the next generation of QM-based scoring functions for HTD toward highly accurate methods for the identification and optimization of small-molecule modulators of pharmaceutically relevant targets.

## Data Availability Statement

The datasets generated for this study are available on request to the corresponding author.

## Author Contributions

CC conceived and designed the research, performed simulations, analysis, interpretation, and wrote the paper. MA performed quantum mechanical simulations and enrichment calculations and contributed with interpretation and analysis. All authors approved the manuscript for publication.

## Conflict of Interest

The authors declare that the research was conducted in the absence of any commercial or financial relationships that could be construed as a potential conflict of interest.
